# Enhanced photoconductive response of ZnO thin films with the impact of annealing temperatures on structural and optical properties

**DOI:** 10.1038/s41598-025-02177-7

**Published:** 2025-08-07

**Authors:** Rajkumar C, Arunachalam Arulraj

**Affiliations:** 1 SANKEN (The Institute of Scientific and Industrial Research), The University of Osaka, Osaka, Japan; 2 Department of Electronics and Communication, University of Allahabad, Prayagraj, Uttar Pradesh India; 3 Departamento de Electricidad, Facultad de Ingeniería, Universidad Tecnológica Metropolitana, Ñuñoa, Santiago, 7800002 Chile

**Keywords:** ZnO, Photodetector, Self-powered, Thin films, Photoconductivity, Photocurrent, Energy science and technology, Materials science, Nanoscience and technology, Optics and photonics

## Abstract

Zinc oxide (ZnO) is a versatile material widely used in optoelectronic devices due to its broad bandgap (3.37 eV), high electron mobility, and significant exciton binding energy (60 meV). In this study, ZnO thin films were fabricated on SiO₂/Si substrates via thermal evaporation, followed by annealing at 400 °C and 600 °C to investigate the effect of thermal treatment on their structural, optical, and photoconductive properties. X-ray diffraction (XRD) analysis confirmed the formation of the hexagonal wurtzite ZnO structure, with improved crystallinity observed at higher annealing temperatures. The photoconductivity of the films demonstrated enhanced response times and self-powered behavior, particularly in the sample annealed at 600 °C. These findings highlight the potential of ZnO thin films for fast-response photodetection applications and show that controlled annealing significantly influences photosensitivity.

## Introduction

Zinc oxide (ZnO) is widely utilized in numerous applications, particularly in photodetectors, due to its broad bandgap (3.37 eV), high electron mobility, and significant exciton binding energy (60 meV)^1,2,2–6^. ZnO can be fabricated in various forms, such as thin films, sintered pellets, powders, and single crystals, among which, thin films are mostly used for applications, including photodetectors, catalysis, gas sensors, and environmental engineering. This preference is due to the low production costs, scalability, small size, and compatibility with microelectronics. Several methods are used to prepare ZnO thin films, including sol-gel, spray pyrolysis, sputtering, and evaporation, among which, evaporation is the simplest and cleanest method. It allows easy control of parameters to tailor film properties. Thermal evaporation is notably cheaper than sputtering, in which the target material is expensive, and the deposition rate is slower. Unlike sputtering, which can cause substrate damage due to high-speed atom collisions, thermal evaporation reduces this risk as energy depends on the evaporating material. Thin films exhibit distinct properties compared to bulk materials, where thin-film characteristics are more influenced by surface quality than volume. These properties can be altered through thickness variation, doping, surface treatments, or the creation of multilayer structures. Thin film technology also optimizes raw material usage^7^.

Photoconductivity is a key factor in various industrial and scientific applications, including biological and chemical sensing, surveillance, optical communications, and environmental monitoring^8,9^. Photodetectors find use in military surveillance, flame detection, air quality monitoring, space communication, optical imaging, and industrial quality control^10–13^. While traditional photodetectors are based on crystalline semiconductors like silicon (Si), germanium (Ge), and gallium arsenide (GaAs), these materials face limitations such as restricted design flexibility, optical signal crosstalk, blurring effects, and the need for high-temperature fabrication^13^. Inorganic semiconductors like ZnO, CeO₂, V₂O_5_, ZnS, InSe, CdS, and CdSe offer greater flexibility in nanostructured form, enabling tunable properties and reduced device dimensionality^14–19^. Various studies have explored ZnO’s growth mechanisms and morphology^20–23^, with these materials primarily functioning for UV photodetection. However, many applications demand visible-light detection, driving research into nanostructured semiconductors, particularly metal oxides, to achieve wide-spectrum detection, including the visible range^24–26^. The optical properties of ZnO can be tuned via doping or by forming composites with other semiconductors.

ZnO stands out for its potential in photodetection due to its unique properties like non-toxicity, low cost, efficiency, high-temperature operation, and environmental stability. For this reason, ZnO was selected to fabricate a photodetector, with thermal evaporation chosen for its simplicity and high deposition rate^27^. Various researchers have successfully used thermal evaporation to synthesize ZnO structures, such as nano pushpins, nanotubes, nanobelts, and nanorods, with zinc or ZnO powder as the starting material^28–35^. Annealing is a crucial post-deposition treatment that significantly affects defect states and crystallinity in ZnO thin films. Higher annealing temperatures typically promote the removal of surface defects, such as oxygen vacancies and zinc interstitials, and enhance the alignment of grains, leading to improved crystallinity. However, excessive annealing can result in grain growth, which reduces grain boundary density and alters defect states, potentially diminishing the built-in electric field critical for photoconductive behavior^36–39^.

In this study, ZnO thin films were deposited on a SiO₂/Si substrate via thermal evaporation, then annealed at 400 °C, and 600 °C. The structural, optical, and photoconductivity properties of the films were analyzed, revealing that films annealed at 600 °C exhibited faster response times (Tr = 0.027 s) (27 ms) due to their high crystallinity. The prepared ZnO photoconductor demonstrated a higher response time compared to those reported in previous studies.

## Materials and methods

**Fig. 1:**
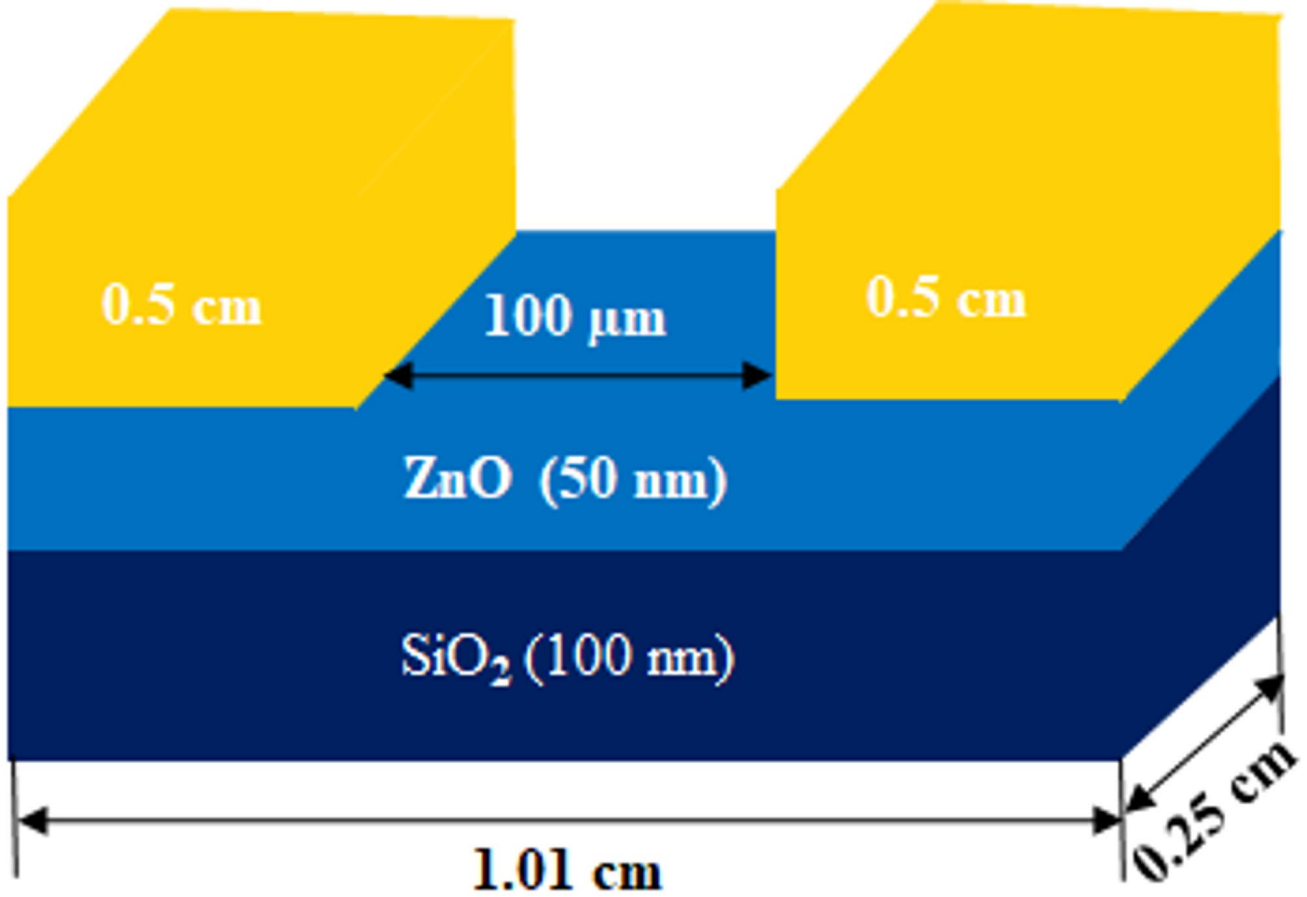
Schematic representation of the fabricated ZnO photoconductor.

The chemicals used in this study were obtained commercially and utilized without any further purification. ZnO powder was synthesized following a method reported in our previous work, using zinc nitrate hexahydrate, diethanolamine, and polyethylene glycol (PEG) as the primary reagents^40^. This ZnO powder was then employed as the target material for fabricating a ZnO thin film on a SiO_2_/Si substrate. The Si (P-type) substrate, with a 100 orientation and a thickness of 0.5 mm, was purchased to prepare the photoconductor. The substrate was cleaned using a solution of H_2_SO_4_ and H_2_O_2_. Initially, a 100 nm SiO_2_ layer was grown on the Si substrate using a nano dry oxidation furnace, heated to 1100 °C for 50 min. After that, the ZnO powder was deposited on the SiO_2_ layer using a thermal evaporation method, with a deposition rate of 5.76 A for the primary current and 12.4 A for the secondary current, under a voltage of 67 V. The deposited ZnO film, with a thickness of 50 nm, was verified using ellipsometry and atomic force microscopy. The unprocessed ZnO was labeled as bare ZnO and ZnOD. The ZnOD sample was divided into three parts and annealed at 400 °C, and 600 °C for two hours each. These annealed samples were labeled as ZnOD400, and ZnOD600, respectively, corresponding to their annealing temperatures. Subsequently, a metal layer of Au:Cr (in an 80:20 ratio) with a thickness of 100 nm was deposited on the ZnO layers using the sputtering method. The exposure area of the ZnO layer (2500 μm in length and 100 μm in width) and the metal contact area (0.5 cm by 0.25 cm) were defined through optical lithography and chemical etching. Finally, a single device, measuring 1.01 cm by 0.25 cm, was sliced from several devices using a cutting technique for photoconductivity applications. Figure 1 illustrates a schematic of the photoconductor structure.

## Results and discussion

**Fig. 2:**
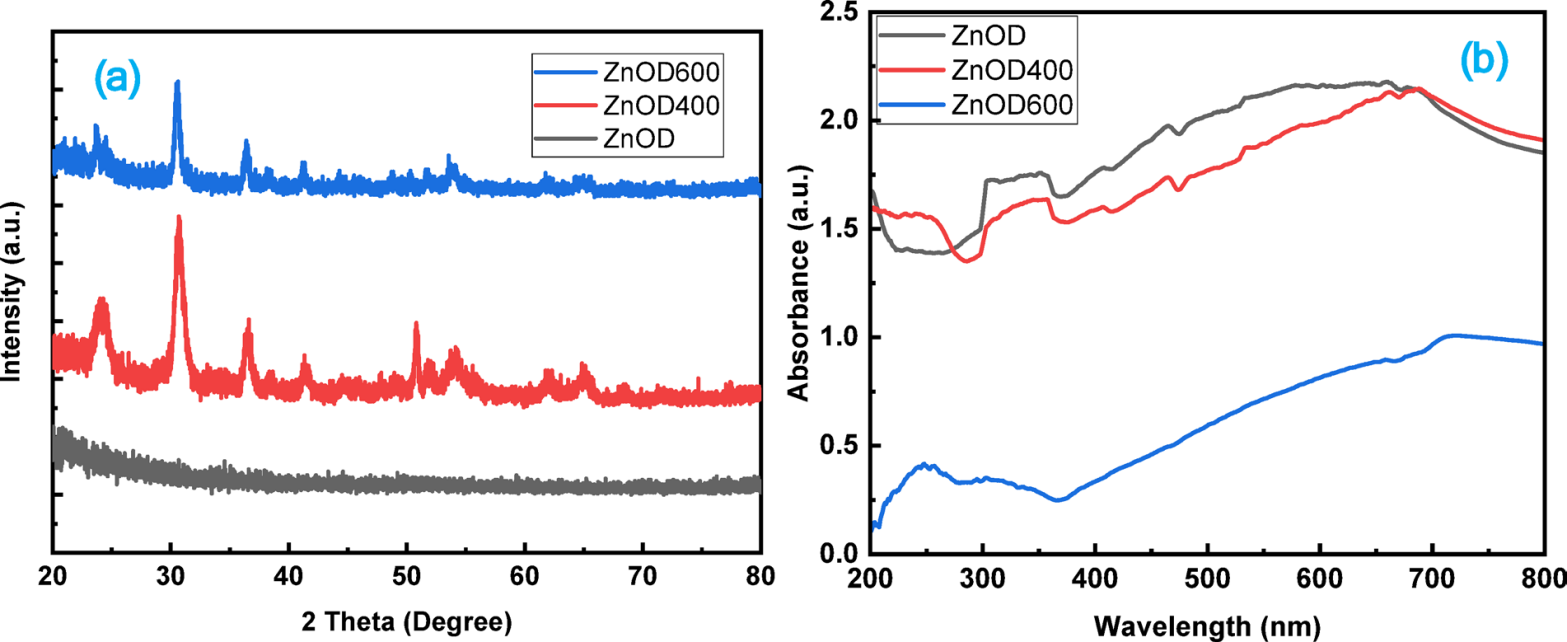
(**a**) XRD and (**b**) UV-visible absorbance spectra of prepared samples.

The crystalline structure of the ZnO thin films deposited on SiO₂/Si substrates was characterized using X-ray diffraction (XRD). Figure 2a shows the XRD patterns of the as-deposited ZnO films and films annealed at various temperatures. Peaks at 2θ values corresponding to 31.77°, 34.42°, and 36.25° were identified, which can be indexed to the (100), (002), and (101) planes of the hexagonal wurtzite ZnO structure, respectively (JCPDS card no. 36-1451). These peaks confirm the successful deposition of ZnO in its crystalline form.

In addition to the characteristic peaks of ZnO, the XRD pattern revealed multiple secondary peaks, suggesting the presence of additional phases. Consistent with our previous findings, annealing the ZnO films at higher temperatures led to the formation of multiphase structures, including an unknown phase and a cubic phase, alongside the primary hexagonal ZnO phase. The appearance of these phases is attributed to the high annealing temperature and potential interactions between the ZnO and the SiO₂/Si substrate. An unknown phase in the annealed films was also observed, particularly at temperatures above 400 °C^41–43^. This unknown phase is often attributed to the high thermal stress experienced by ZnO films during annealing​^40^. As the ZnO samples get down to room temperature after annealing, the secondary phase in ZnO is formed due to tensile stress induced by the lower thermal expansion coefficient of the SiO_2_ matrix, which prevents ZnO from attaining its freestanding crystalline characteristics^44^.

Increasing the annealing temperature from 400 °C to 600 °C enhances the crystallinity of the ZnO films, as indicated by sharper and more intense diffraction peaks. This trend is consistent with the work of Rajkumar et al., where annealing at higher temperatures led to a phase transformation and improved crystalline quality. The crystallite size was estimated using the Debye-Scherrer equation and showed an increase with an annealing temperature (11 nm (400 °C) to 18 nm (600 °C)), which can be attributed to the aggregation of crystallites at elevated temperatures​^40^.

Figure 2b shows the UV-visible absorption spectra of ZnOD, ZnOD400, and ZnOD600 samples, representing ZnO films in the as-deposited state and annealed at 400 °C and 600 °C, respectively. The absorption spectra of the deposited ZnO samples exhibit absorption edges in the 310–357 nm range, indicating the presence of ZnO nanoparticles. All samples show a blue shift compared to their bulk counterpart which typically has an absorption edge around 380 nm. Additionally, absorption edges are also observed in the visible region which may be due to silicon substrate. The absorption edge in the UV region shifts from 325 nm to 250 nm upon annealing, indicating an increase in the optical bandgap of ZnO. This blue shift is primarily attributed to improved crystallinity and a reduction in defect-related states, which previously facilitated lower-energy transitions. Additionally, the absorbance in the visible region gradually decreases with annealing, suggesting a further reduction in deep-level defect states, such as oxygen vacancies and interstitials, which are responsible for sub-bandgap absorption. Since the material consists of a 50 nm ZnO layer deposited on SiO₂/Si (100 nm/0.5 mm), the annealing process likely improves the interface quality and reduces the scattering effects, contributing to increased optical transparency in the visible region. However, full transparency cannot be directly determined from the measurement, as the underlying Si substrate strongly absorbs in the visible and infrared regions. Any reduction in visible absorption observed in ZnO may be masked by the strong optical absorption of Si, making it difficult to isolate the exact transparency level of the ZnO layer alone^45–49^.

**Fig. 3:**
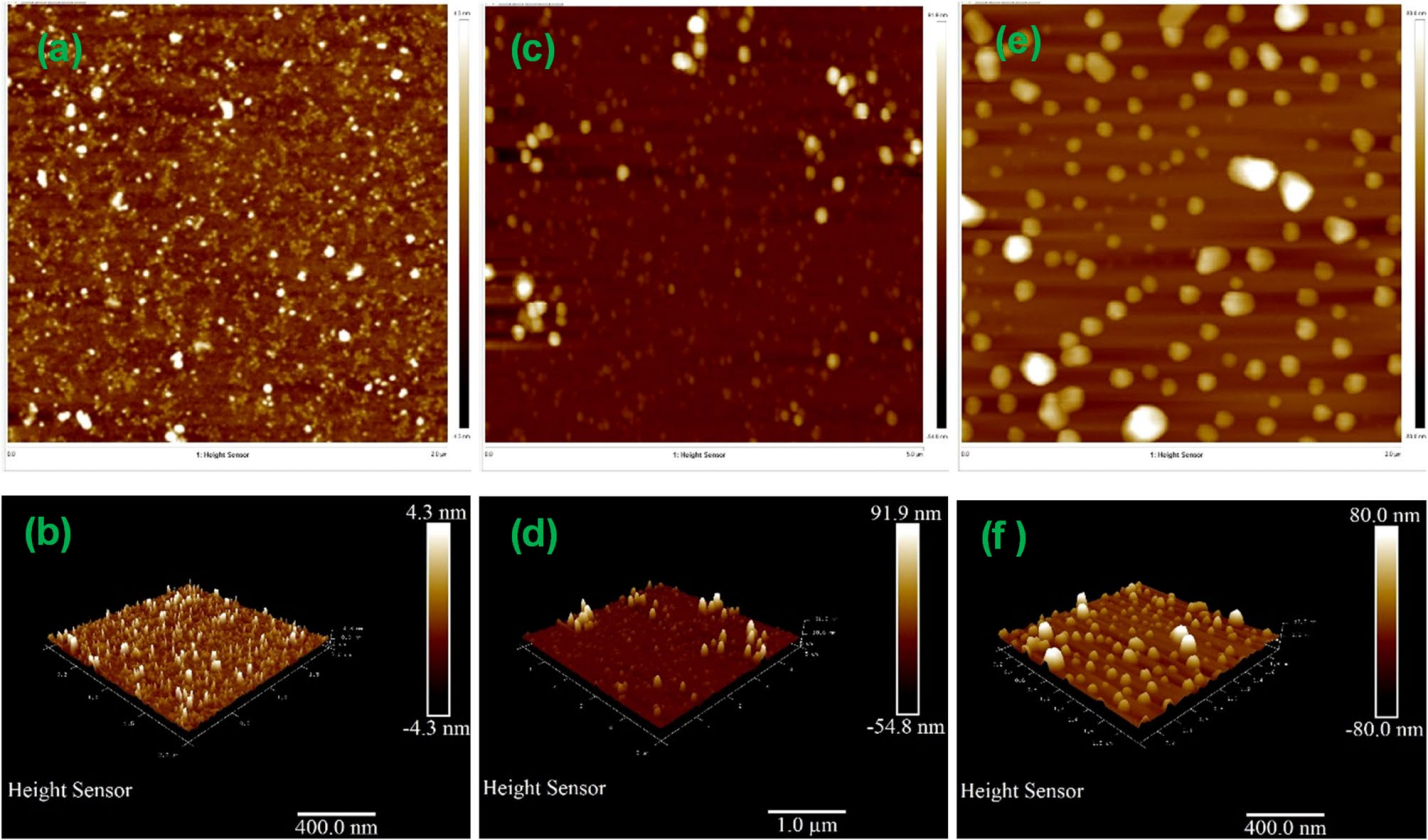
Top view and 3D view AFM images of (**a**,**b**) ZnOD, (**c**,**d**) ZnOD400, and (**e**,**f**) ZnOD600.

Figure 3 shows AFM 2D (a, c,e) and 3D (b, d,f) images of all samples. They show the topography and roughness of all samples. All samples have spherical-like structure. The measured surface roughness of ZnOD, ZnOD400, and ZnOD600 is 0.58 nm, 5.78 nm, and 10.8 nm, respectively. When the annealing temperature increases, the surface roughness is also increased. This increased roughness suggests grain coarsening and surface reconstruction. While a rougher surface can introduce additional surface states that act as recombination centers, thereby reducing carrier lifetime.

**Fig. 4:**
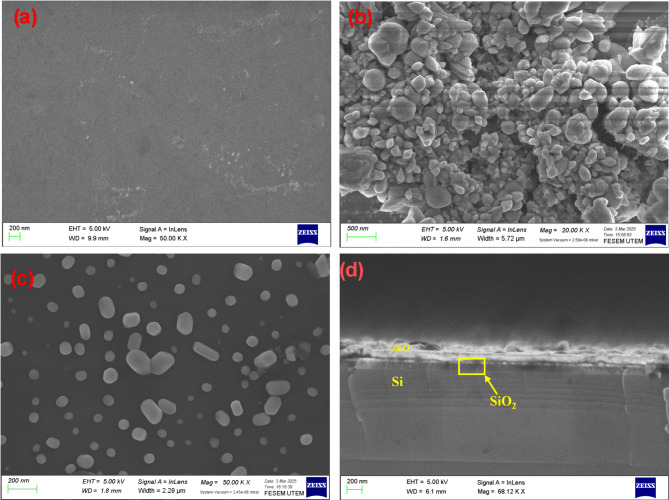
FE-SEM images of (**a**) ZnOD, (**b**) ZnOD400, (**c**) ZnOD600, and (**d**) Cross-sectional FE-SEM image of ZnOD film deposited on the SiO_2_ layer.

Figure 4 shows the FE-SEM images of (a) ZnOD, (b) ZnOD400 and (c) ZnOD600 samples, whereas Fig. 4d presents the cross-sectional FE-SEM image of a ZnO film deposited on the SiO₂ layer. The ZnOD sample (Fig. 4a) exhibits a smooth and uniform ZnO film without distinct grain boundaries, indicating an amorphous structure. This observation is further confirmed by this sample’s XRD (Fig. 2a) and AFM (Fig. 3a) analyses. Figure 4b shows a polyhedral-like structure and agglomerated morphology, with some faceted structures and rough edges rather than perfectly smooth spheres. Figure 4c reveals that the ZnOD600 sample consists of particles with varied shapes and sizes, suggesting a non-uniform growth process during high-temperature annealing. The particles exhibit a mix of faceted and rounded morphologies, with some displaying hexagonal-like structures and some appearing like hexagonal elongated rod-like structures, while others appear more irregular. Hexagonal elongated rod-like structures may form due to the head-to-head fusion of several small nanorods through a process known as ‘oriented attachment,’ where smaller crystals align and merge to form larger crystals. Their distribution suggests partial aggregation, likely influenced by thermal effects during annealing. The observed morphological diversity may result from variations in nucleation and growth dynamics at high temperatures^50–53^.

**Fig. 5:**
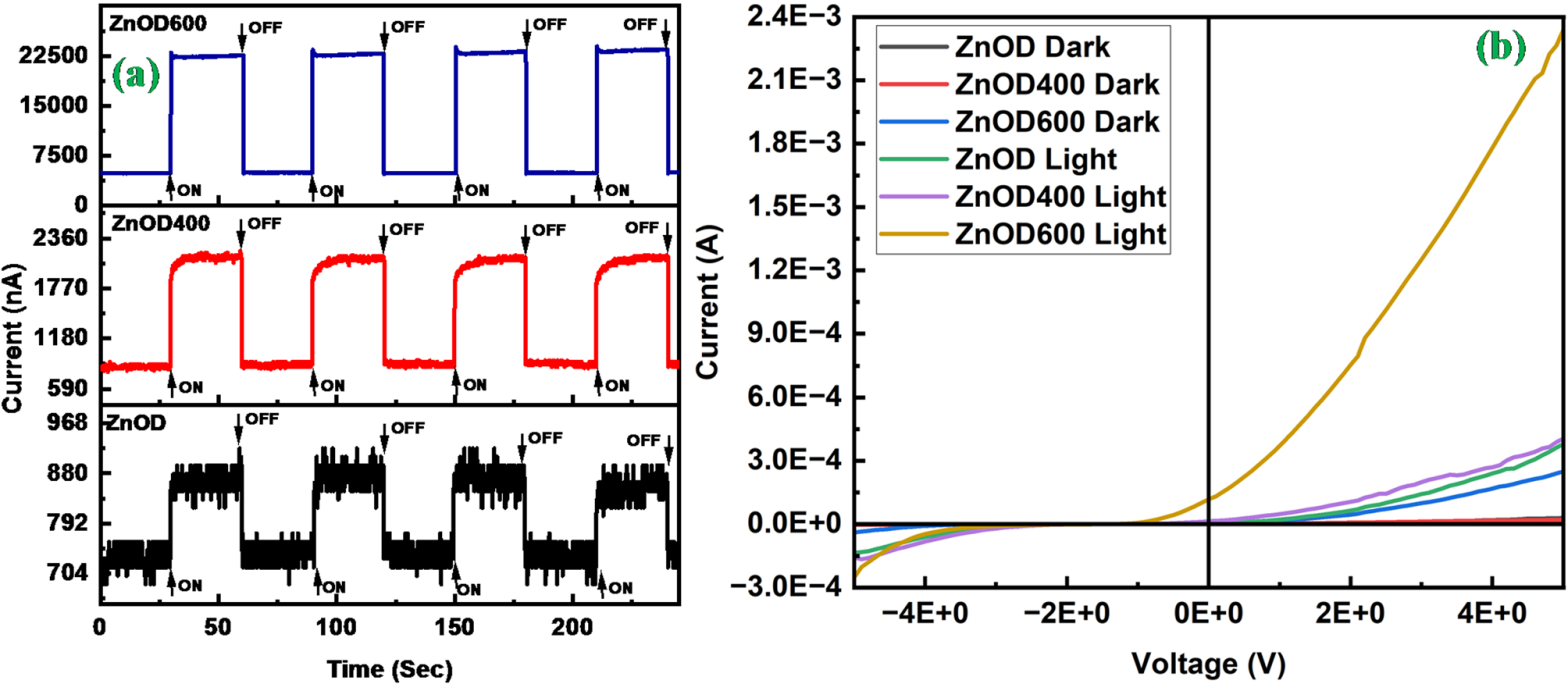
(**a**) Rise and decay curves of photocurrent under the periodic illumination (30 s) for ZnOD, ZnOD400, and ZnOD600 samples. (**b**) The current-voltage (I-V) curves of the prepared devices under dark and illumination.

Figure 5a illustrates the photocurrent response of ZnO photoconductors under periodic illumination. The applied voltage bias during these measurements was 2 V, and the photocurrent was measured in ambient air. A solar simulator (Sol3A Class AAA Solar Simulator) was integrated into the probe station for measurements. It operates at an intensity of 1000 W/m² and employs a xenon arc lamp with a wavelength range of 200–2500 nm. Table 1 shows the measured parameters for the prepared photoconductor. In all samples, upon exposure to light, the current increases sharply due to the photogeneration of electron-hole pairs:

$$\:h\nu\:\to\:{h}^{+}+{e}^{-}$$

In ZnOD and ZnOD400 samples, upon exposure to light, the current increases sharply due to the photogeneration of electron-hole pairs followed by the increased current gradually due to the photodesorption of oxygen molecules which act as surface traps:

$$\:{O}_{2}^{-}+{h}^{+}\to\:{O}_{2}\left(gas\right)$$

When the light is switched OFF, the current decreases due to the recombination of the electrons and holes.

The photocurrent response varies with annealing temperature, with ZnO films annealed at higher temperatures (600 °C) showing faster rise (0.027 s) and decay times (0.066 s) than those annealed at lower temperatures (400 °C) and left unannealed. This improvement in response time is attributed to better crystallinity and smoother surface morphology at higher annealing temperatures, which reduce surface defect states that typically act as recombination centers. The reduction of such defects enhances the mobility and separation efficiency of the carriers, contributing to a faster rise time and higher photocurrent. These findings demonstrate the effectiveness of annealing in improving the performance of ZnO-based photoconductors for fast-response photodetection applications.

**Table 1:** Photocurrent, dark current, responsivity, photosensitivity, rise time, and decay time of all prepared photoconductors.

Sample	Photocurrent (I_pc_)	Dark current (I_dc_)	Responsivity (A/W)	Photosensitivity, $\eta = \frac{{{I_{pc}} - {I_{dc}}}}{{{I_{dc}}}}$	Rise time (10 to 90%) (T_*r*_) (sec)	Decay time (90 to 10%) (T_d_) (sec)
ZnOD	8.64335E^− 07^	7.30124E^− 07^	0.0005	0.183819597	0.30	0.18
ZnOD400	2.1204E^− 06^	8.49378E^− 07^	0.0050	1.496415205	0.8	0.077
ZnOD600	2.23028E^− 05^	4.8601E^− 06^	0.0697	3.58897011	0.027	0.066

The I-V characteristics graph (Fig. 5b) of ZnO-based photoconductors (ZnOD, ZnOD400, ZnOD600) under both dark and light conditions provides insights into the photoconductive and self-powered behavior of these devices. The impact of annealing is evident in the enhanced photocurrent behavior, particularly in the ZnOD600 sample, which exhibits the highest photocurrent under UV illumination. The increased photocurrent after annealing is attributed to defect formation, such as oxygen vacancies, which act as charge-trapping sites, improving charge carrier separation and mobility when the device is exposed to UV light. The I-V graph indicates that all samples, including the annealed ones, exhibit a photocurrent at 0 V under illumination, confirming their self-powered nature. The self-powered behavior stems from the internal electric fields generated by the ZnO layer’s intrinsic properties, such as the built-in electric fields at the metal-semiconductor junction formed by the Au:Cr contact layer deposited via sputtering. These fields facilitate the movement of photogenerated carriers without external bias, as demonstrated by the non-zero current at 0 V. The structure defined through optical lithography ensures that the active area of ZnO is exposed efficiently, contributing to the observed self-powered characteristics. Thus, the combination of careful material processing, such as annealing and precise device fabrication, enhances photoconductivity and enables self-powered operation, particularly in the optimized ZnOD600 sample.

The built-in electric field in ZnO-based photodetectors is optimized at an annealing temperature of 600 °C due to the balanced generation of oxygen vacancies and the enhancement of crystal quality at this temperature. Annealing plays a crucial role in modulating the defect density, crystal orientation, and grain structure of the ZnO film, all of which directly influence the strength of the built-in electric field. At 600 °C, the energy provided is sufficient to promote the formation of a high density of oxygen vacancies defects that are essential for increasing the n-type carrier concentration. These vacancies create donor states that contribute to the overall charge carrier population, enhancing the electric field within the Schottky junction (ZnO/Au) and thus improving the photodetector’s self-powered operation. This optimal defect concentration supports a strong built-in electric field by maintaining an efficient carrier separation and transport mechanism under illumination.

If the annealing temperature is lower, such as at 400 °C, the thermal energy is insufficient to significantly increase the formation of oxygen vacancies or to improve the crystal quality to the extent needed for a robust electric field. As a result, the ZnO film exhibits fewer charge carriers and a less effective electric field. On the other hand, annealing at temperatures higher than 600 °C can lead to grain growth, which reduces the density of grain boundaries—important sites that help stabilize defects and contribute to the electric field. Additionally, over-annealing may cause oxygen vacancies to migrate or recombine, reducing the defect density that supports the built-in electric field. These effects result in a diminished electric field at higher annealing temperatures due to the loss of the optimal balance between defect density and crystal integrity.

At 600 °C, the ZnO film achieves a state where crystallinity is significantly improved without excessive grain growth, and the density of active grain boundaries is maintained. The grain boundaries serve as sites for defect stabilization and play a role in enhancing the electric field by contributing to charge trapping and separation. This controlled defect environment and structural optimization enable a strong electric field, making the 600 °C annealing condition ideal for maximizing the photoconductive response and self-powered functionality of the ZnO-based photodetector. Thus, the annealing temperature of 600 °C is identified as the critical parameter for achieving the best balance between defect formation, grain boundary density, and crystallinity, resulting in the highest built-in electric field and improved device performance^6,8,9,54,36,38,55^.

**Fig. 6:**
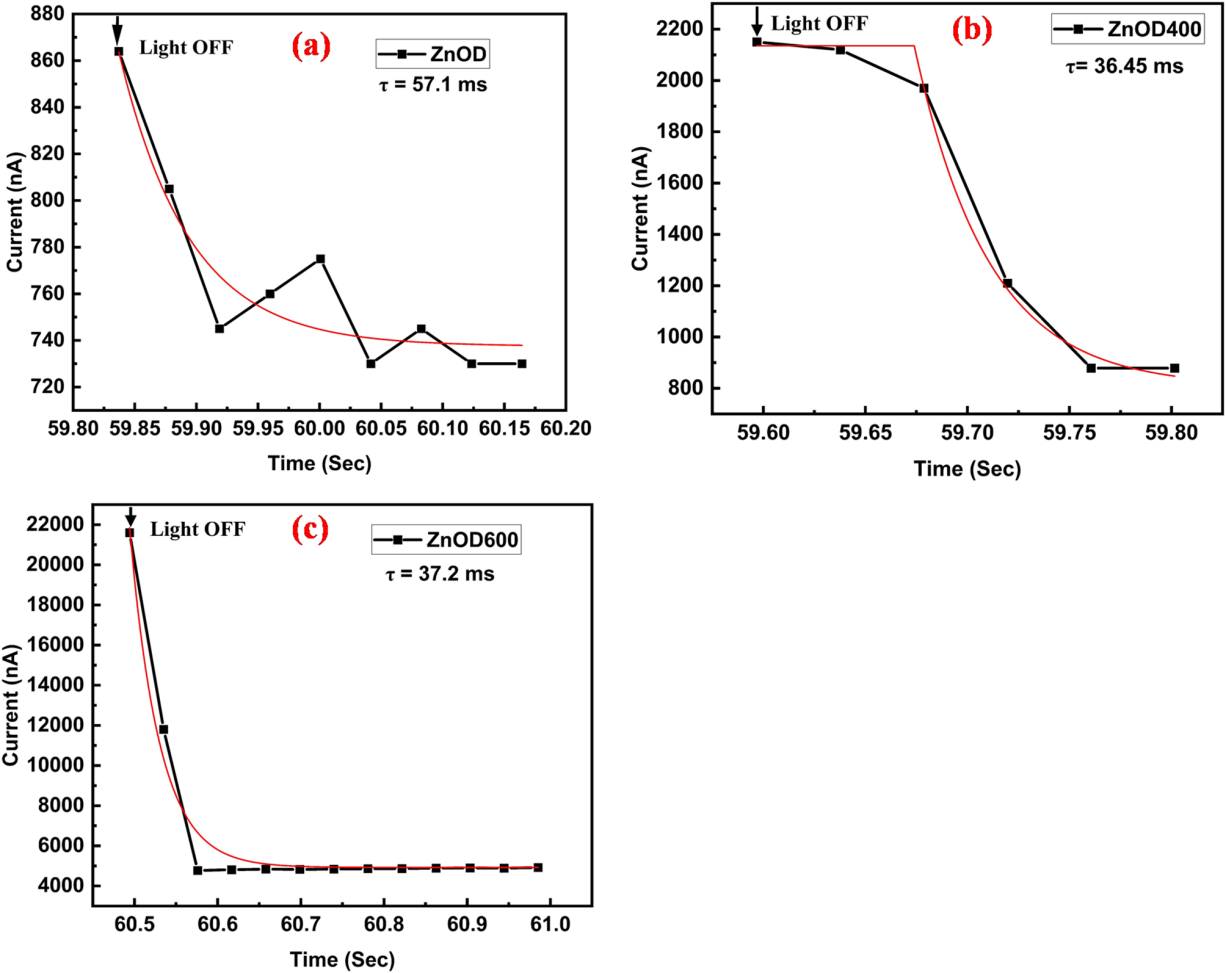
Carrier lifetime of (**a**) ZnOD, (**b**) ZnOD400, and (**c**) ZnOD600, extracted by exponential fitting of the decay portion of the photocurrent response after the light is switched off, as shown in Fig. 5a.

Figure 6 shows the carrier lifetime of (a) ZnOD, (b) ZnOD400, and (c) ZnOD600, extracted by exponential fitting of the decay portion of the photocurrent response after illumination was turned off. The decay of ZnOD and ZnOD600 was modeled using a single-exponential decay function:

$$\:\text{I}\left(\text{t}\right)={I}_{0}+\text{A}\text{*}{e}^{-(\text{t}-{\text{t}}_{0})/{\uptau\:}}$$

where $\:\text{I}\left(\text{t}\right)\:$is the transient current, $\:{I}_{0}\:$is the current when the light is switched off, A is the decay amplitude, and τ is the carrier lifetime, $\:{\text{t}}_{0}$ is the time when exponential decay begins.

In contrast, the ZnOD400 sample exhibited delayed decay behavior, characterized by a plateau prior to recombination, and was therefore fitted using a delayed exponential function that accounts for a time offset T_d_ before the exponential decay begins:

$$\:\text{I}\left(\text{t}\right)=\left\{\begin{array}{c}{I}_{b},\:\:\:\:\:\:\:\:\:\:\:\:\:\:\:\:\:\:\:\:\:\:\:\:\:\:\:\:\:\:\:\:\:\:\:\:t<{T}_{d}\\\:{I}_{0}+A*\left(1-{e}^{-\frac{\text{t}-{\text{t}}_{0}}{{\uptau\:}}}\right),\:t\ge\:{T}_{d}\end{array}\right.$$

where $\:{I}_{b}$ is the baseline current during the delay, $\:{T}_{d}$ is the delay time before decay begins, A is the amplitude of change (with A < 0 for decay), and τ is the carrier lifetime.

**Table:** 

Sample	Lifetime (τ)	Observation
ZnO	63.5 ms	Gradual decay; slight fluctuations due to noise
ZnOD400	36.45 ms	Delayed exponential decay: current initially remains constant before decay begins
ZnOD600	37.2 ms	Clean and fast exponential decay

The extracted carrier lifetimes were 63.5 ms (ZnOD), 36.45 ms (ZnOD400), and 37.2 ms (ZnOD600), with high fitting accuracy (R² ≈ 0.96–0.99). The longer lifetime observed in the unannealed ZnOD sample is attributed to a higher density of deep-level traps or possible amorphous structural components, which slow down recombination by temporarily trapping carriers. Annealing at 400 °C and 600 °C appears to improve crystallinity, reducing trap density and leading to faster recombination dynamics, as reflected in the shorter lifetimes of ZnOD400 and ZnOD600. The delayed decay in ZnOD400 further suggests the presence of shallow traps or slow detrapping processes, which slightly prolong the carrier dynamics before initiating recombination. These observations highlight the influence of post-deposition thermal treatment on the recombination behavior of ZnO-based films and their suitability for optoelectronic applications. The secondary phase of annealed samples shows a short carrier lifetime in ZnO which may be due to introducing trap states, recombination centers, and structural inhomogeneities.

This increased roughness suggests grain coarsening and surface reconstruction. While a rougher surface can enhance light scattering and potentially improve optical absorption, it may also introduce additional surface states that act as recombination centers, thereby reducing carrier lifetime. Additionally, increased surface roughness can raise the metal–semiconductor contact resistance, possibly influencing device efficiency. Despite these concerns, the transient response and carrier lifetime remain favorable, indicating a balance between light management and recombination dynamics.

## Conclusion

In this study, ZnO thin films were successfully deposited on SiO₂/Si substrates using thermal evaporation, followed by annealing at various temperatures (400 °C, and 600 °C) to evaluate their structural, optical, and photoconductive properties. X-ray diffraction (XRD) analysis confirmed the formation of the hexagonal wurtzite ZnO structure, with increased crystallinity observed at higher annealing temperatures. UV-visible absorption spectra and photoconductivity measurements demonstrated that annealing significantly influences the response times and self-powered behavior of the films. Notably, the ZnO thin film annealed at 600 °C exhibited the fastest response times and the highest photocurrent, making it the most optimized condition for photodetector performance. The results indicate that controlled annealing is critical in tuning the properties of ZnO thin films for enhanced photodetection applications. These findings contribute valuable insights into the development of ZnO-based photodetectors and underscore the importance of annealing conditions in optimizing device performance. Future work could focus on further optimizing these conditions or exploring other dopants and deposition methods to expand the application potential of ZnO thin films.

## Data Availability

The datasets used and/or analyzed during the current study are available from the corresponding author upon reasonable request.
